# Chronic back problems and labor force participation in a national population survey: impact of comorbid arthritis

**DOI:** 10.1186/1471-2458-13-326

**Published:** 2013-04-10

**Authors:** Lauren Churcher, Christina H Chan, Elizabeth M Badley

**Affiliations:** 1 Arthritis Community Research and Evaluation Unit, Toronto Western Research Institute, University Health Network, 399 Bathurst Street, MP10-322, Toronto, ON M5T 2S8, Canada; 2 Dalla Lana School of Public Health, University of Toronto, Toronto, Canada

**Keywords:** Arthritis, Back problems, Employment, Comorbidity

## Abstract

**Background:**

Back problems and arthritis are common chronic conditions, while having back problems is a frequent reason for lost work time. The objective of this study was to investigate employment status amongst individuals who report having both back problems and arthritis, compared to having either condition alone.

**Methods:**

We analyzed data from the 2007/2008 Canadian Community Health Survey (ages 25–64, n = 79,719). Respondents who reported neither having worked in the past 12 months nor the past week were coded as not currently employed. Those reported being permanently unable to work were considered to be out of the labor force. Log-Poisson regressions, adjusting for socio-demographic and lifestyle factors, were used to estimate risks for being not currently employed or being out of the labor force for 5 mutually exclusive groups of chronic conditions: arthritis and back problems, back problems, arthritis, any other chronic conditions, and no chronic conditions.

**Results:**

12.7% of respondents reported being not currently employed and 2.9% being out of the labor force. 5.8% of respondents reported both arthritis and back problems, while 16.1% reported back problems and 7.3% arthritis. The back problems and arthritis group had the highest risk of not being currently employed. The risk was higher for men (PR = 1.90; 95% CI = 1.58, 2.29) than for women (PR = 1.31; 95% CI = 1.18, 1.46). Risks of being permanently unable to work were also the greatest for those with comorbid back problems and arthritis.

**Conclusions:**

There is a need for a reappraisal of back problems as a cause of work disability to account for the possibility of co-occurring arthritis.

## Background

Musculoskeletal disorders are among the most costly to society, and the major types of disorders contributing to these costs are back problems and arthritis [1]. The high cost is due to a combination of high population prevalence and the impact of these disabling conditions on productivity [2,3]. Back problems and arthritis are consistently in the top three most frequently reported health conditions in Western Countries, with population prevalences of 15-20% for each condition in the population aged 15 years and older [4-7]. Having back problems is recognized as one of the most frequent reasons for lost work time, a major cause of long term disability and a great economic burden [8-10]. An analysis of the occupational health supplement of the US National Health Interview Survey found that repeated trouble with back, neck or spine was the most prevalent work-related chronic condition and accounted for 49% of workers’ compensation claims filed [11]. In Ontario, Canada, injuries to the back were the most common source of Workplace Safety and Insurance Board lost time claims, accounting for almost a third of all lost time claims [12]. An Australian study found back problems to be the most commonly reported chronic health condition forcing early retirement [2]. Arthritis is one of the most common causes of long-term disability [4,13], and also a frequent reason for work loss [14,15], although unlike back problems, it is not usually attributed to injury. Numerous studies have shown that individuals with arthritis are at greater risk for work loss, work disability and early retirement [2,3,15-17].

Although the burden of back problems on work-time loss and work productivity is well documented, there is a limited body of evidence describing the impact of comorbidities on employment outcomes for individuals with back problems. Several studies have shown that co-occurring chronic physical and mental conditions increases the risk of work loss and work cutback days compared to having a physical or mental condition alone [18-21]. Current evidence shows that chronic back problems are associated with increased risk of a variety of physical conditions including other musculoskeletal disorders, heart disease, migraines and headaches [22-25], and mental conditions [26-28]. Given its high frequency in the population, it is perhaps not surprising that arthritis has been found to be the most commonly reported comorbidity with back problems [23,24]. Nonetheless, the impact of having co-occurring back problems and arthritis has been little studied.

This study builds on an earlier study examining the impact of co-occurring arthritis and back problems on a range of health indicators. It was found that individuals who reported both conditions had significantly higher risks of reporting activity limitation, fair/poor self-rated health and self-rated mental health, and having more than three doctor consultations in the previous month compared to individuals who reported having arthritis only or back problems only [29]. Building on these findings, we hypothesized that having co-morbid back problems and arthritis would also negatively impact labor force participation over and above the effect of having each condition on its own.

The primary objective of this study was to investigate employment status amongst individuals who reported having arthritis but not back problems, those who reported having back problems but not arthritis, those who reported having both, those who reported having other chronic conditions but neither back problems nor arthritis, and those who reported no chronic conditions. The secondary objective was to determine if any sex differences in employment status existed between these groups, since earlier work has shown gender differences in the relationship between labor force participation and health [30-32].

## Methods

### Study design and setting

Data used in this study were obtained from Cycle 4.1 (2007/2008) of the Canadian Community Health Survey (CCHS), which is a cross-sectional survey administered by Statistics Canada to collect information on health status, health care utilization, and health determinants of Canadians aged 12 years and older. Details on the design and administration of the survey are documented elsewhere [33]. Briefly, the CCHS used a two-stage cluster design to generate a nationally representative sample of respondents: in the first stage, provinces and territories were divided into strata based on geographic and socio-demographic characteristics, with target sample sizes defined for each to ensure representativeness of sample; in the second stage, households were randomly sampled from each stratum. Finally, one person per household was randomly selected to complete the interview. Individuals living on First Nations Reserves, on Crown Lands, in institutions, individuals who are full-time members of the Canadian Forces, and residents of remote regions were excluded. It is estimated that the CCHS covered approximately 98% of the Canadian population aged 12 years and older [33]. Data were collected with computer assisted interviews.

Data were obtained through access to Statistics Canada’s Public Use Microdata File collection, which is available to subscribing institutions for a fee. This study restricted analyses to 2007/2008 CCHS respondents of working age (25–64) to capture the population most likely to be employed.

### Variable definitions

#### Outcome variable (employment status)

Respondents were asked “Have you worked at a job or business at any time in the past 12 months?” to which they answered yes or no. Respondents were also asked about their job status in the week prior to the interview: “Last week, did you work at a job or a business? Please include part-time jobs, seasonal work, contract work, self-employment, baby-sitting and any other paid work, regardless of the number of hours worked,” to which they answered yes, no, or permanently unable to work. They were also asked if they had a job from which they were absent in the week prior to the interview: “Last week, did you have a job or business from which you were absent?”. Individuals who reported being permanently unable to work in the past week were considered as being out of the labor force, even if they reported having worked in the past year. Those who reported not having worked in the past 12 months and not having worked in the past week and not being temporarily absent from a job in the past week were considered not currently employed. Individuals who reported working in the past 12 months and did not report being permanently unable to work in the past week were considered to be in the labor force, although they may have been temporarily absent from work in the past week or may not have been employed in the past week.

#### Independent variable (chronic conditions)

Respondents were asked about the presence of “long-term conditions which are expected to last or have already lasted six months or more and that have been diagnosed by a health professional.” Questions were asked about 17 conditions, which included asthma, arthritis (excluding fibromyalgia), back problems (excluding fibromyalgia and arthritis), high blood pressure, migraine headaches, chronic bronchitis, emphysema, chronic obstructive pulmonary disease (COPD), diabetes, heart disease, cancer, stomach or intestinal ulcers, stroke, urinary incontinence, bowel disorder, mood disorder (depression, bipolar disorder, mania or dysthymia) and anxiety disorder (phobia, obsessive-compulsive disorder, panic disorder). Based on this information, respondents were grouped into five mutually exclusive categories: i) having back problems but not arthritis, ii) having arthritis but not back problems, iii) having arthritis and back problems, iv) having other chronic condition(s) and neither back problems nor arthritis, and v) not having any chronic conditions. Being in groups i, ii or iii does not exclude the possibility of having other comorbid chronic condition(s) in addition to back problems and/or arthritis.

#### Covariates (socio-demographic, lifestyle and health characteristics)

Age, sex and education level were included as covariates in the regressions since they are associated with employment outcomes and risk of chronic conditions [14]. Age was categorized into ten year intervals starting with 25–34 and ending with 55–64. Respondents’ highest levels of education achieved were grouped as “less than secondary”, “secondary graduate”, “some post-secondary” and “post-secondary graduate”. Living arrangements were included since previous work has shown an association between employment and living arrangements for people living with chronic diseases [30,31]. Respondents’ living arrangements were classified into four categories: single and living alone or with non-family, living with a partner, living with children, and other.

Several health and lifestyle variables (BMI, smoking, alcohol consumption, physical activity level) were also included as covariates, since they can also be associated with both employment status and presence of chronic conditions [14]. BMI was calculated using the respondents’ reported weight and height by the following calculation: weight(kg)/height^2^(m^2^). BMI was grouped into three categories: underweight/normal weight (<25 kg/m^2^), overweight (25–29.9 kg/m^2^), and obese (≥30.0 kg/m^2^), in accordance with the World Health Organization’s BMI classification [34]. Pregnant women were excluded from analyses. Based on reported history of cigarette smoking, respondents were categorized as current or former smoker or never smoked. Respondents were categorized as a regular drinker (at least once a month), an occasional drinker (less than once a month), or a nondrinker (did not drink any alcohol in the past 12 months) based on their alcohol consumption in the past 12 months. Physical activity was classified as active, moderately active, or inactive, using Statistics Canada’s Physical Activity Index derived from total daily energy expenditure values calculated for 21 leisure time activities [35].

### Statistical analysis

The distribution of socio-demographic and lifestyle characteristics was calculated for employment outcome variables and individuals reporting back problems only, both arthritis and back problems, arthritis only, and any other chronic conditions. Sample weights provided by Statistics Canada were used to account for the study design and sampling frame.

Overall and sex-stratified log-Poisson regression analyses with bootstrap variance estimations were used to estimate the risks of being not currently employed and risks of being out of the labor force for individuals reporting back problems only, both arthritis and back problems, arthritis only, and any other chronic condition(s), in reference to those who reported not having any chronic conditions. Log-Poisson regression yields a prevalence ratio (PR) whose mathematical computation and interpretation is identical to a relative risk [36,37]. The regression models were adjusted for age, BMI, living arrangement, highest level of education completed, smoking status, alcohol intake and physical activity level. Responses with missing values for any variables were excluded from analyses.

SAS Version 9.2. was used for descriptive analyses, and STATA 12 was used for log-Poisson regressions.

## Results

The household level response rate for this survey was 84.6% and the person-level response rate was 91.7% resulting in a 77.6% response rate at the national level. After excluding individuals that did not meet the age criteria for our study, our sample included responses from 79,719 individuals. The employment status of the population is shown in Table 1. The majority (84.4%) of respondents were in the labor force, of whom 83.7% worked both in the last year and the past week. The 16.3% who did not work in the past week included those who were temporarily away from work and those who were looking for work. No information was given about reasons for being temporarily away from work; these could include vacation and other leave as well as sickness absence. Only a minority (12.7%) of respondents were not currently employed, most frequently women. An additional 2.9% reported being out of the labor force. As might be expected, those who were not currently employed or were out of the labor force were more likely to be older.

**Table 1:** Distribution of socio-demographic and lifestyle characteristics among working-age individuals in Canada based on employment status; 2007/2008 Canadian Community Health Survey, ages 25-64

	**Employment status**		
	**In labor force**	**Not currently employed**	**Out of the labor force**
Estimated number of individuals (1,000)	14,830	2,236	504
Prevalence (%)	84.4	12.7	2.9
Sex (%)			
Male	52.6	27.2	50.5
Female	47.4	72.8	49.5
Age (%)			
25-34	26.3	16.9	7.0
35-44	28.4	16.9	15.2
45-54	28.9	18.9	32.7
55-64	16.4	47.3	45.0
Education (%)			
< Secondary grad	8.5	22.5	34.5
Secondary grad	15.4	19.8	15.3
Some post-secondary	6.7	6.7	10.0
Post-secondary grad	69.5	51.0	40.3
BMI (%)			
Underweight/normal	46.8	48.4	39.2
Overweight	35.6	31.1	32.6
Obese	17.6	20.5	28.2
Living arrangement (%)			
Single, with or without others	17.7	16.3	35.5
Living with partner/spouse	25.2	34.4	28.2
Living with children	45.8	39.1	24.3
Other	11.3	10.3	12.0
Smoking (%)			
Never	34.5	39.0	25.8
Current	25.0	22.4	42.5
Former	40.5	38.7	31.7
Alcohol consumption (%)			
Nondrinker	14.3	31.0	42.3
Regular	70.6	50.0	36.7
Occasional	15.1	19.0	21.0
Physical activity (%)			
Active	23.2	24.1	12.9
Moderately active	25.4	25.3	17.7
Inactive	51.4	50.6	69.3

An estimated 16.1% of the population aged 25–64 years reported having back problems, 7.3% reported having arthritis, 5.8% reported having both arthritis and back problems, with a further 24.8% reporting other chronic condition(s) (Table 2). A greater proportion of those with back problems only were male or in younger age groups, and a smaller proportion was obese compared to those with both back problems and arthritis or arthritis only. Overall the characteristics of these latter two groups were more similar to each other than the back problems only group. A higher proportion of individuals with back problems only were in the labor force than those with both back problems and arthritis or arthritis only. Of all the groups, individuals who reported both back problems and arthritis had the highest proportion reporting being out of the labor force.

**Table 2:** Prevalence and distribution of socio-demographic characteristics among working-age individuals (ages 25–64) back problems, arthritis and back problems, reporting arthritis, and any other chronic conditions; 2007/2008 Canadian community health survey

	**No chronic condition**	**Back problem**	**Back problem + Arthritis**	**Arthritis**	**Other chronic condition(s)**
Estimated number of individuals (1,000)	8,401	2,927	1,061	1,328	4,511
Prevalence (%)	46.1	16.1	5.8	7.3	24.8
Sex (%)					
Male	54.0	51.1	41.4	40.4	45.2
Female	46.0	48.9	58.6	59.6	54.8
Age (%)					
25-34	31.9	22.8	5.5	6.5	21.3
35-44	29.9	29.2	17.1	14.5	24.7
45-54	25.2	29.3	35.2	33.0	28.4
55-64	13.0	18.8	42.1	46.0	25.7
Education (%)					
< Secondary grad	8.6	11.5	20.9	16.9	11.7
Secondary grad	16.0	15.0	17.0	17.1	16.2
Some post-secondary	6.0	7.6	7.0	7.3	7.3
Post-secondary grad	69.4	65.9	55.1	58.7	64.9
BMI (%)					
Underweight/normal	52.7	45.1	37.1	36.3	42.3
Overweight	34.5	35.5	35.1	37.0	34.9
Obese	12.8	19.4	27.8	26.7	22.8
Living arrangement (%)					
Single, with or without others	17.2	17.5	21.9	18.8	18.2
Living with partner/spouse	22.8	25.5	35.2	36.4	28.3
Living with children	47.7	45.9	34.7	34.7	42.5
Other	12.3	11.2	8.2	10.1	11.0
Smoking (%)					
Never	22.7	29.1	24.2	29.1	33.0
Current	36.7	29.5	33.8	25.4	24.7
Former	40.6	41.3	42.0	45.6	42.3
Alcohol (%)					
Nondrinker	69.5	15.8	23.9	20.2	18.2
Regular	14.2	68.9	55.2	61.8	64.6
Occasional	16.4	15.3	20.9	18.0	17.2
Physical activity (%)					
Active	25.2	22.0	20.3	20.7	20.8
Moderately active	25.0	24.8	21.8	24.6	26.6
Inactive	49.8	53.2	58.0	54.7	52.6
Work status (%)					
In labor force	89.8	84.7	64.2	73.9	82.0
Not currently employed	9.7	11.6	21.2	20.7	14.7
Out of the labor force	0.5	3.7	14.6	5.4	3.3

Results from log-Poisson regressions showed that after controlling for socio-demographic and lifestyle factors, the highest risk of not being currently employed was found for the back problems and arthritis group, and this risk was substantially higher than that of most other chronic disease groups (Table 3). Sex-stratified analyses yielded some notable differences between the sexes in terms of predictors of being not currently employed. The risk for the back problems and arthritis group was significantly higher for men than women. Having only back problems was a significant predictor of not being currently employed for men but not for women. The impact of increasing age was greater for men than women. For women, living with a partner and/or children was associated with an increased likelihood of being not currently employed. The reverse was observed for men, who were less likely to be not currently employed if living with a partner and/or children. Having higher levels of education was generally protective in terms of employment status.

**Table 3:** Risk of not being currently employed associated with chronic conditions, adjusting for sociodemographic, health and lifestyle factors, from multivariate log-Poisson regressions*

	**Overall**	**Female**	**Male**
	**N = 71272**	**N = 37697**	**N = 33575**
	**PR (95% CI)**	**PR (95% CI)**	**PR (95% CI)**
Chronic condition			
None	1.00	1.00	1.00
Back problem and arthritis	1.43 (1.30, 1.58)†	1.31 (1.18, 1.46)†	1.90 (1.58, 2.29)†
Back problem	1.13 (1.04, 1.23)†	1.05 (0.95, 1.16)	1.35 (1.15, 1.58)†
Arthritis	1.22 (1.11, 1.33)†	1.22 (1.11, 1.34)†	1.29 (1.08, 1.54)†
Other chronic condition(s)	1.19 (1.11, 1.27)†	1.09 (1.01, 1.17)†	1.41 (1.22, 1.63)†
Sex			
Male	1.00		
Female	2.28 (2.14, 2.43)†		
Age			
25-34	1.00	1.00	1.00
35-44	0.89 (0.80, 0.99)†	0.86 (0.77, 0.95)†	1.02 (0.77, 1.35)
45-54	0.94 (0.84, 1.05)	0.79 (0.70, 0.90)†	1.69 (1.31, 2.19)†
55-64	2.86 (2.60, 3.14)†	2.19 (1.98, 2.42)†	6.35 (5.10, 7.91)†
Education			
< Secondary grad	1.00	1.00	1.00
Secondary grad	0.69 (0.64, 0.75)†	0.68 (0.62, 0.74)†	0.68 (0.56, 0.81)†
Some post-secondary	0.64 (0.57, 0.72)†	0.58 (0.50, 0.67)†	0.78 (0.62, 0.98)†
Post-secondary	0.48 (0.44, 0.52)†	0.44 (0.40, 0.48)†	0.58 (0.51, 0.67)†
BMI			
Underweight/normal	1.00	1.00	1.00
Overweight	0.86 (0.81, 0.92)†	0.89 (0.83, 0.96)†	0.84 (0.75, 0.94)†
Obese	0.96 (0.89, 1.03)	0.95 (0.88, 1.04)	1.04 (0.89, 1.21)
Smoking status			
Never	1.00	1.00	1.00
Current	0.97 (0.90, 1.05)	0.92 (0.84, 1.00)	1.13 (0.95, 1.34)
Former	0.97 (0.91, 1.04)	0.95 (0.88, 1.02)	1.02 (0.87, 1.20)
Alcohol consumption			
Non-drinker	1.00	1.00	1.00
Regular	0.53 (0.49, 0.57)†	0.53 (0.49, 0.58)†	0.56 (0.48, 0.65)†
Occasional	0.68 (0.63, 0.74)†	0.69 (0.63, 0.75)†	0.72 (0.59, 0.88)†
Physical activity			
Active	1.00	1.00	1.00
Moderately active	0.86 (0.80, 0.92)†	0.97 (0.90, 1.06)	0.66 (0.58, 0.76)†
Inactive	0.74 (0.69, 0.79)†	0.84 (0.77, 0.91)†	0.57 (0.50, 0.64)†
Living arrangement			
Single, with/without others	1.00	1.00	1.00
With partner	1.10 (1.02, 1.18)†	1.33 (1.23, 1.45)†	0.72 (0.65, 0.81)†
With children	1.14 (1.05, 1.23)†	1.53 (1.40, 1.67)†	0.50 (0.42, 0.60)†
Other	1.00 (0.88, 1.13)	1.16 (1.01, 1.34)†	0.79 (0.63, 1.00)†

* Values shown are prevalence ratios (PR) with 95% confidence intervals (CI).

† indicates statistical significance of p < 0.05.

N = Analytic sample. Variance estimations were derived using bootstrap weights provided by Statistics Canada to account for sampling design for the CCHS.

Factors found to be associated with being out of the labor force are shown in Table 4. Prevalence ratios for the both back problem and arthritis group were significantly higher than those for all other chronic disease groups, except for arthritis in women. The prevalence ratios for back problems only were not statistically different from those for the arthritis only or other chronic condition groups, overall and in sex stratified analyses. In contrast to the findings for not being currently employed, other predictors of being out of the labor force were similar between the sexes.

**Table 4:** Risk of being out of the labor force associated with chronic conditions, adjusting for sociodemographic, health and lifestyle factors, from multivariate log-Poisson regressions*

	**Overall**	**Female**	**Male**
	**N = 62873**	**N = 31263**	**N = 31610**
	**PR (95% CI)**	**PR (95% CI)**	**PR (95% CI)**
Chronic condition			
None	1.00	1.00	1.00
Back problem and arthritis	19.50 (14.90, 25.52)†	17.32 (11.63, 25.79)†	21.06 (14.80, 29.98)†
Back problem	7.56 (5.75, 9.93)†	6.54 (4.37, 9.78)†	8.53 (5.97, 12.22)†
Arthritis	8.26 (6.14, 11.11)†	8.37 (5.49, 12.76)†	7.49 (4.91, 11.43)†
Other chronic condition(s)	5.72 (4.25, 7.70)†	4.34 (2.86, 6.59)†	7.13 (4.82, 10.55)†
Sex			
Male	1.00		
Female	0.92 (0.80, 1.05)		
Age			
25-34	1.00	1.00	1.00
35-44	2.57 (1.89, 3.49)†	2.22 (1.50, 3.28)†	2.98 (1.79, 4.96)†
45-54	3.84 (2.86, 5.15)†	3.30 (2.25, 4.84)†	4.34 (2.64, 7.13)†
55-64	6.02 (4.35, 8.33)†	4.59 (3.11, 6.78)†	7.84 (4.66, 13.17)†
Education			
Less than secondary grad	1.00	1.00	1.00
Secondary grad	0.54 (0.45, 0.65)†	0.57 (0.44, 0.72)†	0.52 (0.40, 0.67)†
Some post-secondary	0.84 (0.66, 1.06)	1.08 (0.80, 1.46)	0.59 (0.40, 0.87)†
Post-secondary	0.46 (0.40, 0.53)†	0.45 (0.38, 0.54)†	0.48 (0.39, 0.60)†
BMI			
Underweight/normal	1.00	1.00	1.00
Overweight	0.90 (0.77, 1.05)	1.02 (0.82, 1.27)	0.78 (0.62, 0.97)†
Obese	1.08 (0.94, 1.25)	1.09 (0.90, 1.32)	1.06 (0.85, 1.31)
Smoking status			
Never	1.00	1.00	1.00
Current	1.86 (1.59, 2.19)†	1.98 (1.62, 2.41)†	1.72 (1.31, 2.25)†
Former	1.06 (0.91, 1.24)	1.16 (0.94, 1.44)	0.96 (0.74, 1.24)
Alcohol consumption			
Non-drinker	1.00	1.00	1.00
Regular	0.32 (0.28, 0.38)†	0.25 (0.20, 0.31)†	0.41 (0.33, 0.50)†
Occasional	0.65 (0.56, 0.75)†	0.55 (0.46, 0.65)†	0.82 (0.64, 1.04)
Physical activity			
Active	1.00	1.00	1.00
Moderately active	1.05 (0.83, 1.32)	1.13 (0.80, 1.60)	0.99 (0.73, 1.34)
Inactive	1.39 (1.14, 1.69)†	1.40 (1.02, 1.92)†	1.38 (1.05, 1.81)†
Living arrangement			
Single, with/without others	1.00	1.00	1.00
With partner	0.61 (0.54, 0.69)†	0.74 (0.63, 0.86)†	0.49 (0.41, 0.59)†
With children	0.49 (0.40, 0.60)†	0.52 (0.42, 0.65)†	0.44 (0.32, 0.59)†
Other	0.65 (0.51, 0.82)†	0.59 (0.42, 0.83)†	0.66 (0.46, 0.95)†

* Values shown are prevalence ratios (PR) with 95% confidence intervals (CI).

† indicates statistical significance of p < 0.05.

N = Analytic sample. Variance estimations were derived using bootstrap weights provided by Statistics Canada to account for sampling design for the CCHS.

Two sensitivity analyses were performed to examine the robustness of the results. The log-Poisson regressions were replicated with individuals 25–54 years-old to reduce potential effects of early retirement. This showed no substantial difference from the primary analyses [Additional file 1]. Another analysis with missing BMI data coded as a separate category to account for potential bias resulting from non-response and exclusion of pregnant women also showed no difference.

## Discussion

This study showed that having both back problems and arthritis is associated with a greater likelihood of being not currently employed and being out of the labor force, than having either condition alone. To date most of the literature on labor force participation and back problems has not considered the impact of co-occurring arthritis. Our findings give a new perspective on the emphasis on back problems as a major cause of work disability. They suggest that interventions to maintain work participation in those with back problems need to take into account the possibility of co-occurring arthritis. The findings may also help partially explain why interventions to reduce back pain related impact on functioning and absences at work have only met with limited success [38,39]. Common interventions include educational or exercise components that usually focus on biomechanical aspects such as lifting techniques, with the implication that much of low back pain is related to biomechanical factors and injury [40]. If there is indeed a sub-category of back problems involving comorbidity with arthritis that is associated with loss of labor force participation, this may need to be taken into account in the development of preventive strategies. For those with back problems and arthritis, interventions may need to consider that other joints, most likely the knees, hands, feet and hip, might also be affected. In this instance, biomechanical-focused back pain preventive strategies such as bending from the knees rather than the back when lifting may be inappropriate for individuals with knee symptoms. Instead, strategies related to osteoarthritis management, such as self-management, physical activity and, if appropriate, muscle strengthening exercises and weight loss might be needed.

While it is not clear what the back problems and arthritis group represents, the similarity to the arthritis only group in terms of risks of not being in the labor force as well as the profile of socio-demographic and lifestyle characteristics tentatively suggests that a portion of the back problems and arthritis group might represent a contribution from arthritis with spinal involvement, such as osteoarthritis [29,41]. The profile of characteristics of the back problems only group is similar to that for chronic non-specific low back pain [25].

This study supports previous findings on the impact of chronic diseases on labor force participation [2,14,15,42], and suggests that particular attention needs to be paid to back problems and arthritis. Our findings extend previous analyses which showed that the combination of back problems and arthritis was more highly associated with pain and activity limitation and having poor self-rated heath than either condition alone, even after controlling for the effect of other chronic health conditions [29]. It is likely that these higher levels of pain and disability are contributory reasons for the increased risk for individuals with both back problems and arthritis of not being employed or being out of the labor force. The population impact of back problems and arthritis is not trivial as their combined prevalence (29.2%) in the working age population exceeds that of all other chronic conditions taken together (24.8%). Furthermore, the prevalence of co-occuring arthritis and back problem, the group with the highest risk for being not employed or being out of the labor force, is 5.8%, which is comparable to the prevalence of diabetes in our study population (5.1%).

There were some noteworthy differences between the sexes. Generally, the risks of non-participation in the labor force associated with chronic diseases were higher for men than for women. In addition, there was a gendered pattern of associations between employment status and living arrangements, where living with a partner and/or living with dependent children was associated with a higher likelihood of not being currently employed for women but a lower likelihood for men. This was consistent with earlier findings [30,31] and may reflect the effect of a proportion of women being not employed due to child-care and other domestic responsibilities. On the other hand, both men and women were less likely to report being out of the labor force when they also report living with a partner and/or living with dependent children. This could reflect support from those at home to facilitate being able to work.

A possible confounder not accounted for in our study was type of occupation. Back problems and/or arthritis could differentially affect that risk of not being in the labor force for those with physically demanding jobs [43]. Data on type of occupation in the CCHS were only available for individuals who were currently working and were therefore not available for our analyses. However, descriptive analyses stratified by age showed no clear pattern of occupation type by disease condition [data provided in Additional file 2].

A major strength of this study is the use of data from a national population-based study with broad coverage and a large, representative sample. There are also a number of limitations. First, chronic conditions were ascertained by self-report of health professional diagnoses and was not validated; however, validation studies have shown that self-reported questions about arthritis are a reasonably reliable and economic means to estimate population prevalence of arthritis and other chronic conditions [44,45]. Second, the outcome of “not currently employed” relates to a long-standing absence from the workforce of a year or more. This means this study does not capture short time sickness absence from the workforce, and thus underestimates the impact of chronic conditions on work loss. Third, the questions about back and arthritis problems in the survey referred to chronic problems which had lasted or were expected to last six months or more. This means that acute and/or episodic back problems from which the respondent expected rapid recovery may not have been captured. This may mean that the impact of back problems on work loss may be underestimated in this study. It is feasible that the impact of back problems in particular is greater for shorter work absences [3]. Fourth, individual with back problems and arthritis could also have other chronic conditions which could contribute to their employment status. In a study using data collected from Australians aged 45 to 64, Schofield and colleagues found that among individuals reporting back problems, increasing number of comorbid conditions reported was associated with increasing risk of being out of the labor force [46]. When we controlled for comorbidities in a sensitivity analysis, the risks for being out of the labour force and not being currently employed associated with back problems and arthritis were attenuated in comparison to the primary analysis, but remained statistically significant [see Additional file 3]. This showed that while the number of comorbid conditions is an important predictor of employment status, the specific combination of back problems and arthritis poses a heightened risk for negative employment outcomes. Fifth, employment status was self-reported and was not validated against any formal employment records. Finally we included those who were away from work in the previous week in the in the labor force category. If some of these absences were due to back problems, arthritis, or other chronic conditions we may have underestimated the impact of these conditions on labor force participation.

## Conclusions

Our findings of an increased impact of co-occurring back problems and arthritis on non-participation in the labor force suggests a need for re-appraisal of the role of back problems as a cause of work-loss and emphasizes the need to move towards an approach that recognizes the effect of co-occurring conditions in population health research on employment. In particular, the co-occurrence of arthritis and back problems may deserve additional attention considering its prevalence and impact on health and employment status.

## Competing interests

The authors declare that they have no competing interests.

## Authors’ contributions

EMB conceived of and supervised the study. LC and CHC obtained data and conducted statistical analyses. All authors contributed to the interpretation of the results, helped draft the manuscript and contributed important intellectual content. All authors read and approved the final manuscript.

## Pre-publication history

The pre-publication history for this paper can be accessed here:

http://www.biomedcentral.com/1471-2458/13/326/prepub

## Supplementary Material

Additional file 1 Results from sensitivity analysis: Log-Poisson regressions with study population aged 25-44. Click here for file

Additional file 2 **Prevalence of chronic conditions among different occupational groups, by age group.** Data from the Canadian Community Health Survey 2007/2008. Click here for file

Additional file 3 Results from Log-Poisson regressions adjusted for comorbidity. Click here for file
