# Supplementary material for: Chronic back problems and labor force participation in a national population survey: impact of comorbid arthritis
Source: BMC Public Health. 2013 Apr 10;13:326. doi: 10.1186/1471-2458-13-326 (PMC3626871; doi:10.1186/1471-2458-13-326)
Supplement: Additional file 1 — Results from sensitivity analysis: Log-Poisson regressions with study population aged 25-44. [file 1471-2458-13-326-S1.doc]

Additional file 1. Results from sensitivity analysis: Log-Poisson regressions with study population aged 25-44.

Table 1. Risk of not being currently employed associated with chronic conditions, adjusting for sociodemographic, health and lifestyle factors, from multivariate log-Poisson regressions.*

|  | All  N = 52081 | Female  N = 27185 | Male  N = 24896 |
| --- | --- | --- | --- |
|  | PR (95% CI) | PR (95% CI) | PR (95% CI) |
| Chronic condition |  |  |  |
| None | 1.00 | 1.00 | 1.00 |
| Back problem and arthritis | 1.78 (1.50, 2.10)† | 1.44 (1.19, 1.73)† | 3.19 (2.33, 4.36)† |
| Back problem | 1.08 (0.96, 1.22) | 1.06 (0.93, 1.21) | 1.25 (0.94, 1.65) |
| Arthritis | 1.25 (1.04, 1.51)† | 1.22 (0.99, 1.49) | 1.47 (0.98, 2.22) |
| Other chronic condition(s) | 1.08 (0.98, 1.19) | 1.04 (0.93, 1.17) | 1.24 (0.97, 1.58) |
| * Regression adjusted for age, sex, body mass index, education level, smoking status, alcohol consumption, physical activity, and living arrangement. Values shown are prevalence ratios (PR) with 95% confidence intervals (CI).  † indicates statistical significance of p < 0.05.  N = Analytic sample. Variance estimations were derived using bootstrap weights provided by Statistics Canada to account for sampling design for the CCHS. | | | |

Table 2. Risk of being out of the labor force associated with chronic conditions, adjusting for sociodemographic, health and lifestyle factors, from multivariate log-Poisson regressions.*

|  | Overall  N = 48730 | Female  N = 24218 | Male  N = 24512 |
| --- | --- | --- | --- |
|  | PR (95% CI) | PR (95% CI) | PR (95% CI) |
| Chronic condition |  |  |  |
| None | 1.00 | 1.00 | 1.00 |
| Back problem and arthritis | 27.39 (19.56, 38.35)† | 27.88 (17.34, 44.84)† | 25.55 (16.05, 40.65)† |
| Back problem | 9.29 (6.50, 13.28)† | 9.37 (5.60, 15.67)† | 9.28 (5.91, 14.57)† |
| Arthritis | 10.96 (7.21, 16.67)† | 11.32 (6.29, 20.39)† | 10.66 (6.15, 18.48)† |
| Other chronic condition(s) | 6.49 (4.55, 9.27)† | 5.63 (3.37, 9.41)† | 7.63 (4.82, 12.07)† |
| * Regression adjusted for age, sex, body mass index, education level, smoking status, alcohol consumption, physical activity, and living arrangement. Values shown are prevalence ratios (PR) with 95% confidence intervals (CI).  † indicates statistical significance of p < 0.05.  N = Analytic sample. Variance estimations were derived using bootstrap weights provided by Statistics Canada to account for sampling design for the CCHS. | | | |
