# Supplementary material for: Chronic back problems and labor force participation in a national population survey: impact of comorbid arthritis
Source: BMC Public Health. 2013 Apr 10;13:326. doi: 10.1186/1471-2458-13-326 (PMC3626871; doi:10.1186/1471-2458-13-326)
Supplement: Additional file 2 — Prevalence of chronic conditions among different occupational groups, by age group. Data from the Canadian Community Health Survey 2007/2008. [file 1471-2458-13-326-S2.doc]

Additional file 2. Prevalence of chronic conditions among different occupational groups, by age group. Data from the Canadian Community Health Survey 2007/2008.

|  |  | Management/ Art, education | Business/ Finance | Sales, services | Trades/ Transportation | Primary industry |
| --- | --- | --- | --- | --- | --- | --- |
|  | None | 61.9 | 60.9 | 57.5 | 63.8 | 66.4 |
| 25-34 | Arthritis | 1.7 | 1.2 | 2.4 | 1.7 | 2.1 |
|  | Back | 13.9 | 15.1 | 16.2 | 16.2 | 12.7 |
|  | Both | 1.0 | 0.6 | 1.7 | 1.1 | 1.5 |
|  | Others | 21.6 | 22.2 | 22.2 | 17.2 | 17.4 |
|  |  |  |  |  |  |  |
| 35-44 | None | 53.7 | 52.1 | 51.1 | 54.5 | 56.3 |
|  | Arthritis | 3.2 | 3.6 | 4.6 | 3.8 | 4.3 |
|  | Back | 16.9 | 15.9 | 18.7 | 20.6 | 17.0 |
|  | Both | 2.7 | 2.6 | 4.6 | 3.7 | 1.9 |
|  | Others | 23.6 | 26.0 | 21.1 | 17.4 | 20.6 |
|  |  |  |  |  |  |  |
| 45-54 | None | 44.6 | 43.6 | 40.1 | 44.8 | 44.1 |
|  | Arthritis | 8.0 | 7.2 | 10.8 | 7.9 | 6.6 |
|  | Back | 17.5 | 16.1 | 15.8 | 18.5 | 16.4 |
|  | Both | 4.5 | 6.7 | 8.2 | 7.6 | 5.9 |
|  | Others | 25.4 | 26.5 | 25.1 | 21.2 | 27.1 |
|  |  |  |  |  |  |  |
| 55-64 | None | 35.5 | 31.6 | 31.3 | 32.3 | 30.1 |
|  | Arthritis | 12.7 | 16.5 | 17.4 | 15.6 | 12.2 |
|  | Back | 15.9 | 13.0 | 12.5 | 16.3 | 15.1 |
|  | Both | 7.0 | 8.9 | 11.3 | 9.4 | 10.7 |
|  | Others | 29.0 | 30.0 | 27.6 | 26.5 | 32.0 |
|  |  |  |  |  |  |  |
|  |  |  |  |  |  |  |
|  |  |  |  |  |  |  |
