# Supplementary material for: Chronic back problems and labor force participation in a national population survey: impact of comorbid arthritis
Source: BMC Public Health. 2013 Apr 10;13:326. doi: 10.1186/1471-2458-13-326 (PMC3626871; doi:10.1186/1471-2458-13-326)
Supplement: Additional file 3 — Results from Log-Poisson regressions adjusted for comorbidity. [file 1471-2458-13-326-S3.doc]

Additional file 3. Results from Log-Poisson regressions adjusted for comorbidity.

Table 1. Risk of not being currently employed associated with chronic conditions, adjusting for sociodemographic, health and lifestyle factors and comorbidity, from multivariate log-Poisson regressions.*

|  | All  N = 71272 | | | Female  N = 37697 | | | Male  N = 33575 | | |
| --- | --- | --- | --- | --- | --- | --- | --- | --- | --- |
|  | PR (95% CI) | | | PR (95% CI) | | | PR (95% CI) | | |
| Chronic condition |  | | |  | | |  | | |
| None | 1.00 |  |  | 1.00 |  |  | 1.00 |  |  |
| Back problem and arthritis | 1.03 | (0.93, | 1.14) | 1.06 | (0.95, | 1.19) | 0.99 | (0.82, | 1.21) |
| Back problem | 0.97 | (0.88, | 1.07) | 0.93 | (0.83, | 1.05) | 1.06 | (0.89, | 1.28) |
| Arthritis | 1.16 | (1.04, | 1.29)† | 1.10 | (0.98, | 1.25) | 1.39 | (1.12, | 1.72)† |
| Other chronic condition(s) | 1.09 | (1.02, | 1.18)† | 1.02 | (0.94, | 1.11) | 1.24 | (1.06, | 1.44)† |
| Comorbidity** |  |  |  |  |  |  |  |  |  |
| 0 | 1.00 |  |  | 1.00 |  |  | 1.00 |  |  |
| 1 or 2 | 1.25 | (1.17, | 1.34)† | 1.18 | (1.09, | 1.29)† | 1.41 | (1.23, | 1.63)† |
| 3+ | 1.52 | (1.38, | 1.69)† | 1.39 | (1.24, | 1.56)† | 2.16 | (1.76, | 2.65)† |
| * Regressions were adjusted for age, sex, body mass index, education level, smoking status, alcohol consumption, physical activity, and living arrangement. Values shown are prevalence ratios (PR) with 95% confidence intervals (CI).  ** Number of chronic conditions in addition to index condition(s)  † indicates statistical significance of p < 0.05.  N = Analytic sample. Variance estimations were derived using bootstrap weights provided by Statistics Canada to account for sampling design for the CCHS. | | | | | | | | | |

Table 2. Risk of being out of the labor force associated with chronic conditions, adjusting for sociodemographic, health and lifestyle factors, and comorbidity, from multivariate log-Poisson regressions.*

|  | All  N = 62873 | | | Female  N = 31263 | | | Male  N = 31610 | | |
| --- | --- | --- | --- | --- | --- | --- | --- | --- | --- |
|  | PR (95% CI) | | | PR (95% CI) | | | PR (95% CI) | | |
| Chronic condition |  | | |  | | |  | | |
| None | 1.00 |  |  | 1.00 |  |  | 1.00 |  |  |
| Back problem and arthritis | 3.54 | (2.54, | 4.95)† | 3.25 | (2.06, | 5.12)† | 3.43 | (2.15, | 5.45)† |
| Back problem | 3.36 | (2.46, | 4.58)† | 2.62 | (1.67, | 4.11)† | 4.03 | (2.67, | 6.07)† |
| Arthritis | 6.40 | (4.63, | 8.85)† | 5.15 | (3.27, | 8.10)† | 7.45 | (4.81, | 11.53)† |
| Other chronic condition(s) | 3.64 | (2.60, | 5.09)† | 2.71 | (1.77, | 4.14)† | 4.49 | (2.81, | 7.15)† |
| Comorbidity ** |  |  |  |  |  |  |  |  |  |
| 0 | 1.00 |  |  | 1.00 |  |  | 1.00 |  |  |
| 1 or 2 | 2.49 | (2.03, | 3.06)† | 2.39 | (1.82, | 3.15)† | 2.61 | (1.96, | 3.47)† |
| 3+ | 6.98 | (5.62, | 8.67)† | 6.97 | (5.19, | 9.34)† | 6.67 | (4.82, | 9.23)† |
| * Regressions were adjusted for age, sex, body mass index, education level, smoking status, alcohol consumption, physical activity, and living arrangement. Values shown are prevalence ratios (PR) with 95% confidence intervals (CI).  ** Number of chronic conditions in addition to index condition(s)  † indicates statistical significance of p < 0.05.  N = Analytic sample. Variance estimations were derived using bootstrap weights provided by Statistics Canada to account for sampling design for the CCHS. | | | | | | | | | |
